# Surface Characteristics and Reliability Assessment of Duplex-Surface-Treated SKD61 Die-Casting Mold Steel

**DOI:** 10.3390/ma19214581

**Published:** 2026-10-29

**Authors:** Dong-O Kim, Min-Hyeok Park, Yeongsu Ha, Won-Jon Yang, Sung Kang, Chang-Hui Lee, Soo-Sung Oh, Jae-Yeon Kim, Jai-Won Byeon

**Affiliations:** 1 Department of Materials Science and Engineering, Seoul National University of Science & Technology, Seoul 01811, Republic of Korea; 2 Korea Institute of Materials Science, Changwon 51508, Republic of Korea; 3 Research Institute of Industrial Science & Technology, Pohang 37673, Republic of Korea; 4 Geumseong Heat Treatment Co., Ltd., Pyeongtaek 17800, Republic of Korea

**Keywords:** SKD61, sulfonitriding, microshot peening, magnetic Barkhausen noise, wear resistance, thermal-shock fatigue

## Abstract

**Highlights:**

- Duplex microshot peening and sulfonitriding formed FeS-containing compound layers on SKD61 die-casting mold steel.
- Compared with gas nitriding, sulfonitriding reduced counterbody and total wear, attributed to FeS.
- Erosion-resistance against molten aluminum improved with increasing compound-layer thickness.
- In thermal-shock tests, thicker compound layers tended to reduce crack density but increase crack length.
- Barkhausen-noise coercivity and remanence decreased as the compound layer thickened, enabling nondestructive assessment.

**Abstract:**

Die-casting molds undergo wear, erosion, and repeated thermal loading, which progressively degrade their surfaces and reduce their service life. Compared with conventional gas nitriding, sulfonitriding can improve wear resistance; however, its effects on other reliability-related properties, particularly erosion and thermal-shock fatigue, and the relationships between these properties and the compound-layer characteristics remain insufficiently understood. In this study, duplex surface treatments combining sulfonitriding with three microshot peening conditions, resulting in surface-roughness values of Rz 14, 18, and 22 μm, were applied to SKD61-based (FDAC) die-casting mold steel. The duplex-treated layers were evaluated based on their microstructure, hardness, wear resistance, erosion resistance, and thermal-shock-fatigue resistance, followed by a nondestructive assessment using Barkhausen noise. Sulfonitriding produced FeS-containing compound layers and reduced the wear of the counterbody and the tribo-pair compared with the results for gas nitriding, which is consistent with the solid-lubrication effect of FeS. The erosion resistance increased with the compound-layer thickness. The thermal-shock-fatigue tests indicated a preliminary trade-off between crack initiation and crack propagation as a function of the compound-layer thickness. These results demonstrate that sulfonitriding modifies the surface performance of SKD61 and that the compound-layer thickness is a key parameter governing mold reliability. Moreover, Barkhausen-noise analysis offers a nondestructive assessment of the compound-layer thickness after treatment.

## 1. Introduction

Industry trends, such as automotive lightweighting and electric-vehicle development, have driven a rapid increase in the development and use of metal components. This growing demand for components has resulted in an increase in die-casting production volume. A critical issue in the die-casting process is the service life of the steel molds. Die-casting molds are continuously exposed to cyclic thermal-shock stress resulting from repeated direct contact with high-temperature molten metal, followed by rapid cooling [1,2,3]. These repeated rapid heating–cooling cycles induce localized thermal stress on the mold surface, causing thermal-fatigue damage [4]. Under these conditions, two failure mechanisms are primarily responsible for shortening the mold life. The first is erosion, resulting from the chemical reaction with and mechanical wear caused by the molten-metal flow [3,5,6]. The second is heat checking, in which repeated thermal fatigue generates surface microcracks [1,2,3]. Failure analyses of H13/SKD61 mold steels based on field data confirmed that erosion and heat checking act in combination as the primary damage mechanisms governing mold life [7,8,9].

To address these issues, mold life has been improved by the formation of a hardened layer on the mold steel surface, and a duplex surface treatment combining microshot peening and nitriding has been extensively studied as a representative approach. Applying shot peening prior to nitriding induces plastic deformation and grain refinement at the surface, which promotes nitrogen diffusion and results in a thicker, harder compound layer than that resulting from single-step nitriding [10,11,12,13]. The resulting compound layer simultaneously improves wear resistance and thermal-fatigue resistance, and recent studies have actively investigated the microstructural and property control of the compound layer by adjusting the peening and nitriding conditions [14,15,16,17].

The service life of a surface-treated mold is governed by the degree of degradation of the surface-treatment layer [1,2,3]. Conventional methods for evaluating this degradation rely on destructive evaluation approaches, such as measuring the surface hardness, or direct observation of the cross-section using scanning electron microscopy. These methods are fundamentally limited because they are destructive and require the sectioning of the specimens. This limitation is particularly problematic for mold components with complex geometries, for which destructive hardness testing is often impractical, emphasizing the need for a nondestructive quality-evaluation technique [18]. Therefore, an evaluation technique that can nondestructively and accurately assess the thickness and integrity of the surface-treatment layer after treatment, for example, during quality inspection, is required.

Moreover, applying sulfonitriding instead of gas nitriding can improve wear resistance through the solid-lubrication effect of the FeS phase formed within the compound layer [19,20,21,22]. Recent studies have also investigated duplex surface treatments combining sulfonitriding with shot peening [23].

The previous studies have four main limitations. First, most studies on duplex treatments combining peening and nitriding have dealt with gas or plasma nitriding, and few have examined sulfonitriding combined with peening [23,24,25]. Second, most studies have evaluated a single property, such as hardness or wear resistance, whereas die-casting molds fail by a combination of wear, erosion and heat checking [7,8,9]. Third, these properties have rarely been related to a common microstructural factor such as the compound-layer thickness. Fourth, the treated layers are usually evaluated destructively, and nondestructive studies on nitrided steels have mainly addressed the case depth and hardness of the diffusion layer rather than of the compound layer [18,26,27].

Therefore, a comprehensive evaluation of the reliability of duplex-treated mold steel requires the simultaneous consideration of wear resistance, erosion resistance, and thermal-shock-fatigue resistance, rather than merely individual evaluations. Additionally, the identification of the key microstructural factors, namely, compound-layer thickness and composition, governing these properties is required. Furthermore, a method capable of nondestructively assessing these microstructural factors would contribute to the quality control of die-casting molds.

In our previous study, we demonstrated that a duplex surface treatment combining microshot peening (0.5 mm ball, 5 MPa, 180 s) with sulfonitriding produces a thicker and harder compound layer than that for gas nitriding alone [23]. That study reported the microstructure, residual stress and hardness for a single peening condition. The present study extends the peening conditions, evaluates the erosion and thermal-shock-fatigue resistance, relates these properties to the compound-layer thickness, and examines whether this thickness can be assessed nondestructively.

In this study, duplex surface treatments combining sulfonitriding with three microshot peening conditions, resulting in surface-roughness values of Rz 14, 18, and 22 μm, were applied to SKD61-based die-casting mold steel and compared with the conventional gas-nitriding duplex treatment. The hardened surface layers were analyzed by cross-sectional SEM observation and XRD. The hardness, wear resistance, erosion resistance and thermal-shock-fatigue behavior (heat-checking resistance) were evaluated as key factors governing mold reliability. Furthermore, the feasibility of assessing the compound-layer thickness nondestructively by magnetic Barkhausen-noise analysis was examined.

## 2. Materials and Methods

### 2.1. Materials and Surface Treatment

The specimen material was FDAC (Hitachi Metals, Ltd., Tokyo, Japan), a pre-hardened, free-machining hot-work die steel based on JIS SKD61, supplied in the quenched-and-tempered (QT) condition (39.7–41.6 HRC). Its chemical composition (ladle analysis from the manufacturer’s test certificate) is listed in Table 1. Compared with standard SKD61, FDAC contains added S (0.10–0.15 wt%) for machinability and lower specified V (0.30–0.80 wt%). The duplex surface treatment consisted of microshot peening followed by gas nitriding or sulfonitriding. Microshot peening was performed to produce mean surface-roughness values of Rz 14, 18, and 22 μm. The surface roughness of each specimen was measured using a Mitutoyo SurfTest SJ-410 instrument (Mitutoyo, Kawasaki, Japan). Microshot peening was carried out using steel balls with diameters of 0.3–0.7 mm, at peening pressures of 3–7 MPa, peening times of 30–300 s, and peening distances of 100–250 mm. The detailed microshot peening conditions are presented in Table 2. Gas nitriding and sulfonitriding were performed under four conditions, in which the treatment time, nitriding temperature, and gas composition were varied. The gas-nitriding conditions are denoted as GN, and the sulfonitriding conditions are denoted as SN. The details of the GN and SN conditions are listed in Table 3. GN and SN were carried out as commercial processes in a gas-nitriding furnace at Geumseong Heat Treatment Co., Ltd. (Pyeongtaek, Republic of Korea); GN was performed in NH_3_, and SN in NH_3_ with H_2_S. The detailed process parameters, including the temperature program and the gas composition and flow rates, are proprietary to the heat-treatment company; Table 3, therefore, gives the temperature and time ranges from the process records. The treated conditions are additionally characterized by the resulting compound- and diffusion-layer thicknesses and phases (Section 3.1).

The duplex treatments were applied to plates of 153 mm × 53 mm × 6.5 mm, from which specimens of 35 mm × 35 mm × 5 mm were cut for the microstructural analysis, XRD, hardness and Barkhausen-noise measurements. The wear specimens were cut from the remaining parts of the treated plates and tested against ø12 mm S45C disks, the erosion specimens were 40 mm × 40 mm × 20 mm, and the thermal-shock-fatigue specimens were cylinders 43 mm in diameter and 60 mm in height. The specimens were taken from the same treatment batch as the one used in our previous study [23], in which only the non-peened and P2-peened specimens were analyzed.

### 2.2. Microstructural and Property Analysis

The duplex-treated specimens were sectioned and mounted in epoxy resin for cross-sectional microstructural observations. The mounted specimens were polished using #220–2000 SiC paper, followed by a 1.0–0.05 μm Al_2_O_3_ suspension, and they were etched with a 2% Nital solution for 5–15 s. The microstructures were examined using optical microscopy (OM; BA310Met, Motic, Xiamen, China) and scanning electron microscopy (SEM; TESCAN VEGA3, Tescan, Brno, Czech Republic). The phases present in the hardened surface layer were identified by XRD (X’Pert PRO MPD, PANalytical, Almelo, Netherlands). The microhardness values of the specimens were measured on their cross-sections using a micro-Vickers hardness tester (FV-800, Future Tech, Kawasaki, Japan) under a load of 100 gf for a dwell time of 15 s. The hardness depth profiles were measured with one indentation at each depth; the first indentation was made 15 μm below the surface, and the hardness of the compound layer itself was not measured. The compound-layer thickness was measured on cross-sectional SEM images as the local distance from the surface to the compound/diffusion-layer boundary, perpendicular to the local surface; the diffusion-layer thickness was measured in the same way. For each condition, 10 measurements were taken for each layer, and the mean and standard deviation are reported. The surface residual stress of representative specimens (P2-GN and P2-SN1–SN3) was measured by the XRD sin^2^ψ method using a portable X-ray stress analyzer (Xstress 3000 G2R, Stresstech, Jyväskylä, Finland) at the Gyeongbuk Hybrid Technology Institute (Yeongcheon, Republic of Korea), as in our previous study [23].

The wear resistance of the hardened surface layers was evaluated using a high-frequency friction/wear tester (Plint TE77 Auto, Phoenix Tribology Ltd., Kingsclere, UK). All wear tests were conducted without lubrication under ambient conditions (25 ± 2 °C, 60 ± 1% relative humidity). The wear resistance was quantified from the weight loss of the specimens, as shown in Equation (1), where M_s_ and M_f_ denote the initial weight of the wear specimen and its weight after the wear test, respectively. The tests were carried out at an accredited laboratory (Daegu Mechatronics & Materials Institute, Daegu, Republic of Korea). The treated specimen was used as the fixed plate and slid against a ø12 mm S45C disk (counterbody), in area contact. One test was performed for each condition, and the weight losses of both the specimen and the counterbody were measured. The test conditions are listed in Table 4.

(1) $$\mathrm{Wear}\;\mathrm{Weight}\;\mathrm{Loss}\;(mg)=M_{s}-M_{f}$$

**Figure 1:**
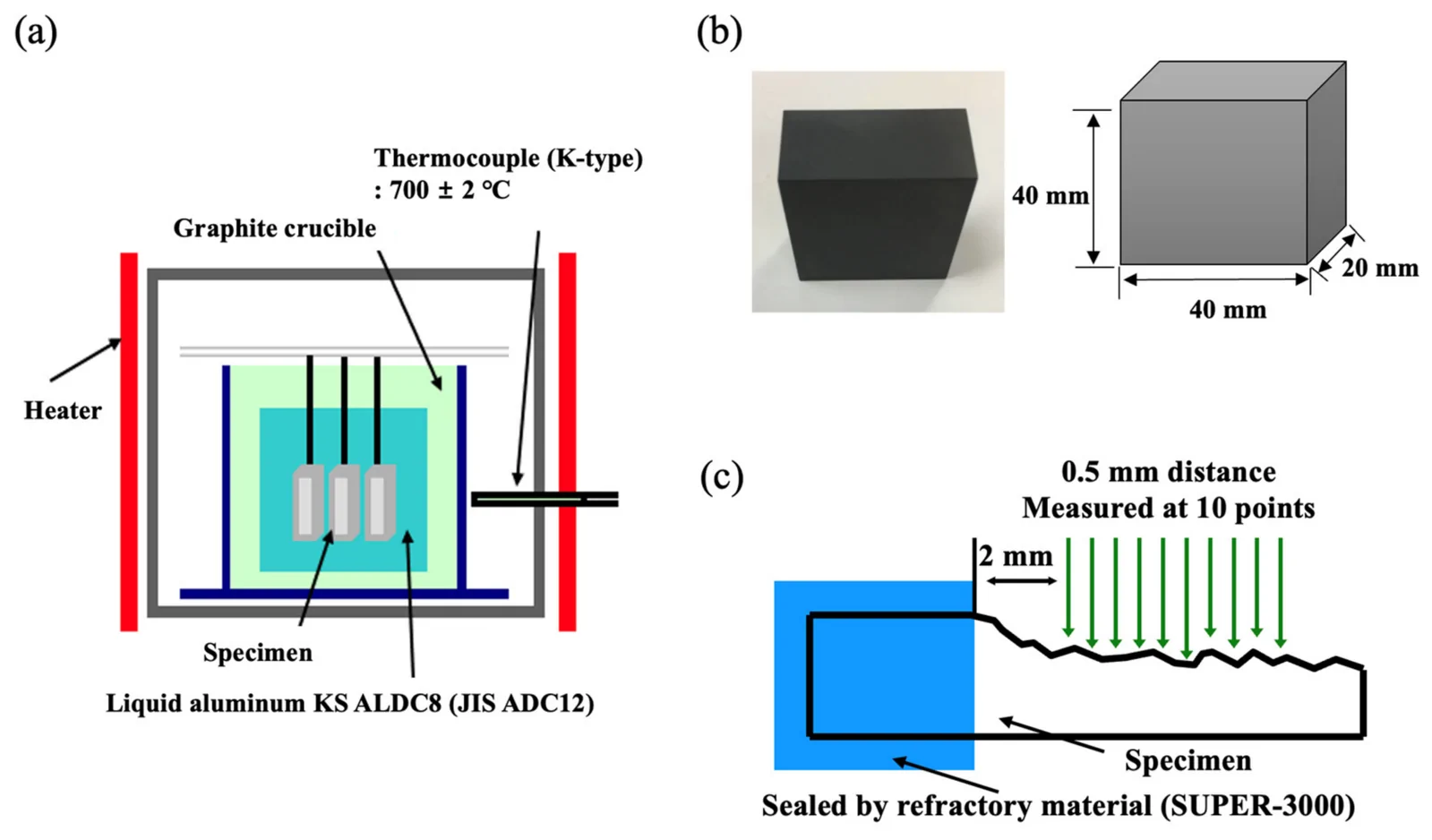
Erosion test: (**a**) schematic of the immersion test in molten aluminum; (**b**) specimen and its dimensions; (**c**) measurement of the melt-out depth.

Figure 1 shows the test setup and evaluation method used to assess the erosion resistance of the duplex-treated specimens. Specimens measuring 40 mm × 40 mm × 20 mm were sealed at one end with a refractory material and then immersed for 43 h in molten aluminum at 700 °C (conforming to KS ALDC8 [28] and JIS ADC12 [29]), as shown in Figure 1a. The melt-out depth at the surface exposed to the molten aluminum was measured at ten points spaced 0.5 mm apart, and the average melt-out depth was defined as the erosion-resistance parameter.

**Figure 2:**
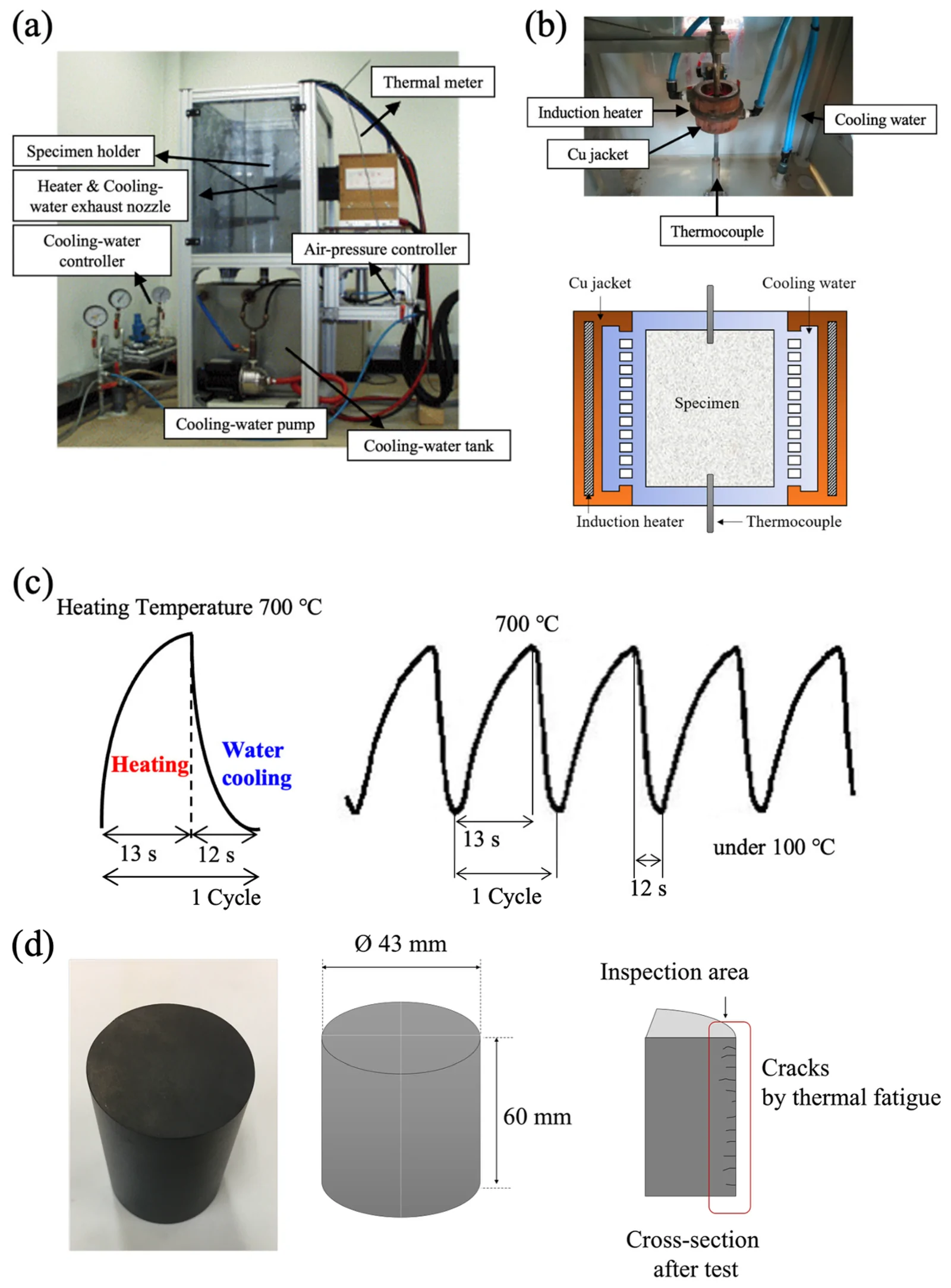
Thermal-shock-fatigue test setup: (**a**) apparatus, (**b**) induction heating and cooling equipment, (**c**) cycle definition, and (**d**) specimen geometry.

Die-casting molds are exposed to molten aluminum at approximately 700 °C followed by rapid cooling; therefore, the thermal-shock-fatigue behavior of the specimens was also evaluated. Figure 2 shows the dedicated test apparatus, specimen-mounting schematic, and test procedure used to evaluate thermal-shock-fatigue behavior (heat-checking resistance). As shown in Figure 2b, the specimen temperature was rapidly raised to 700 °C within 13 s using induction heating and then quenched to below 100 °C within 12 s using cooling water. As illustrated in Figure 2c, this heating–cooling sequence was defined as one cycle, and a total of 3000 cycles were applied. Cylindrical specimens 43 mm in diameter and 60 mm in height were used, as shown in Figure 2d, and the crack length on the specimen surface was measured on the specimen cross-section after testing. One specimen was tested for each condition. All cracks along 61–69 mm of the cross-section (154–306 cracks per specimen) were measured, and the number of cracks per unit length, the average crack length and the maximum crack length were determined.

### 2.3. Barkhausen-Noise Analysis

**Figure 3:**
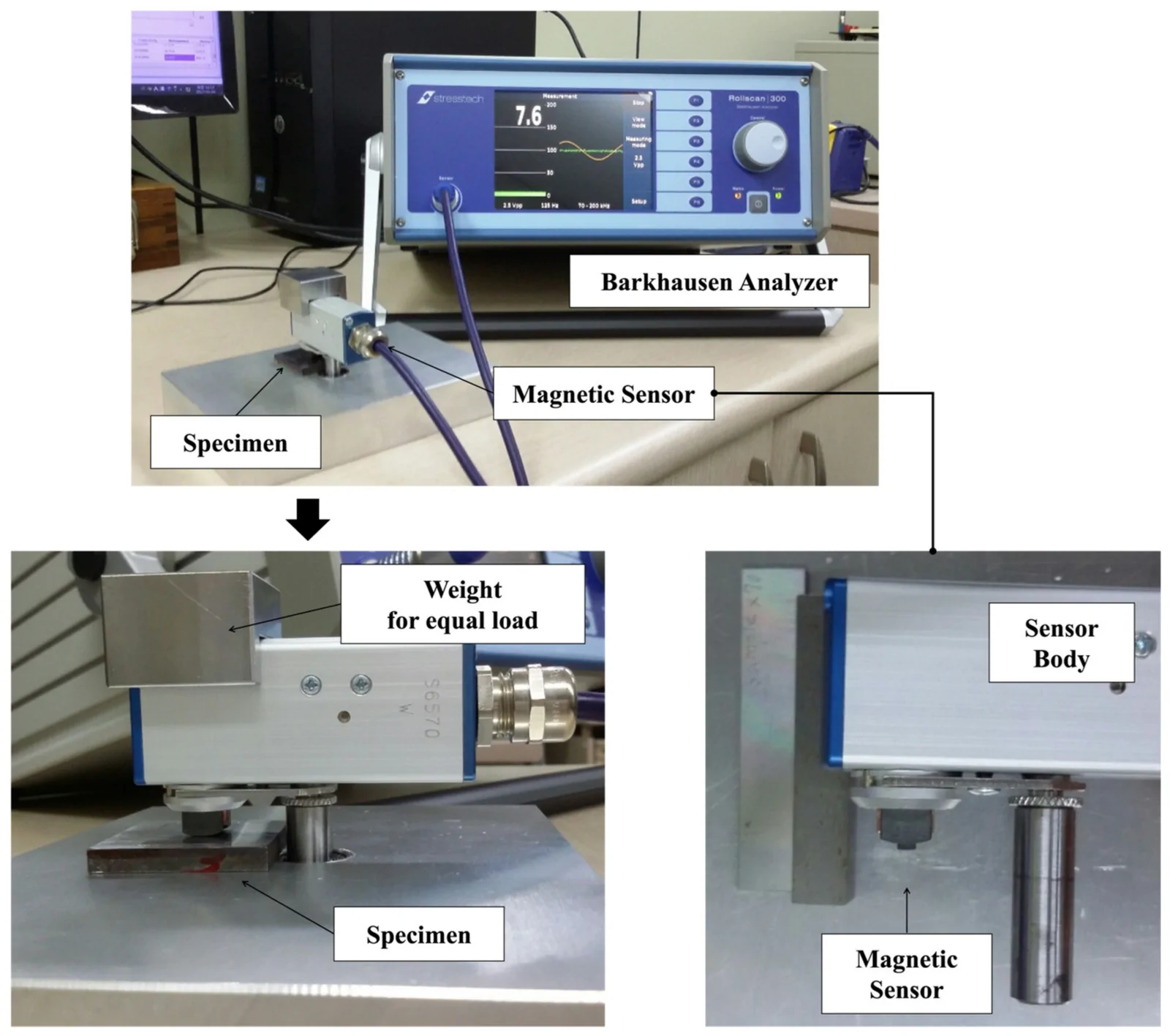
Photograph of the magnetic Barkhausen-noise signal-analysis equipment.

Figure 3 shows the setup used to evaluate the magnetic parameters of the duplex-treated specimens using a digital Barkhausen-noise analyzer (Rollscan 300, Stresstech Group, Jyväskylä, Finland). Barkhausen noise was measured using an SX-18 sensor under sinusoidal magnetization at 125 Hz and a magnetizing voltage of 3 Vpp; the signal was analyzed in the frequency range of 70–200 kHz. Coercivity and remanence were calculated using µScan 600 software (Stresstech Group) from the integral of the Barkhausen-noise bursts plotted against the magnetizing current. These quantities are therefore given in the relative units of the analyzer (a.u.) and do not correspond to the coercive field and remanent induction of a conventional hysteresis loop. For this analyzing frequency range, the information depth of the Barkhausen-noise signal in hardened steel is of the order of 0.1 mm [26,27], which covers the compound layer and part of the diffusion layer. A constant load was applied to the sensor using identical weights, and each value is the average of five measurements at randomly selected locations on each specimen.

## 3. Results and Discussion

### 3.1. Microstructure

**Figure 4:**
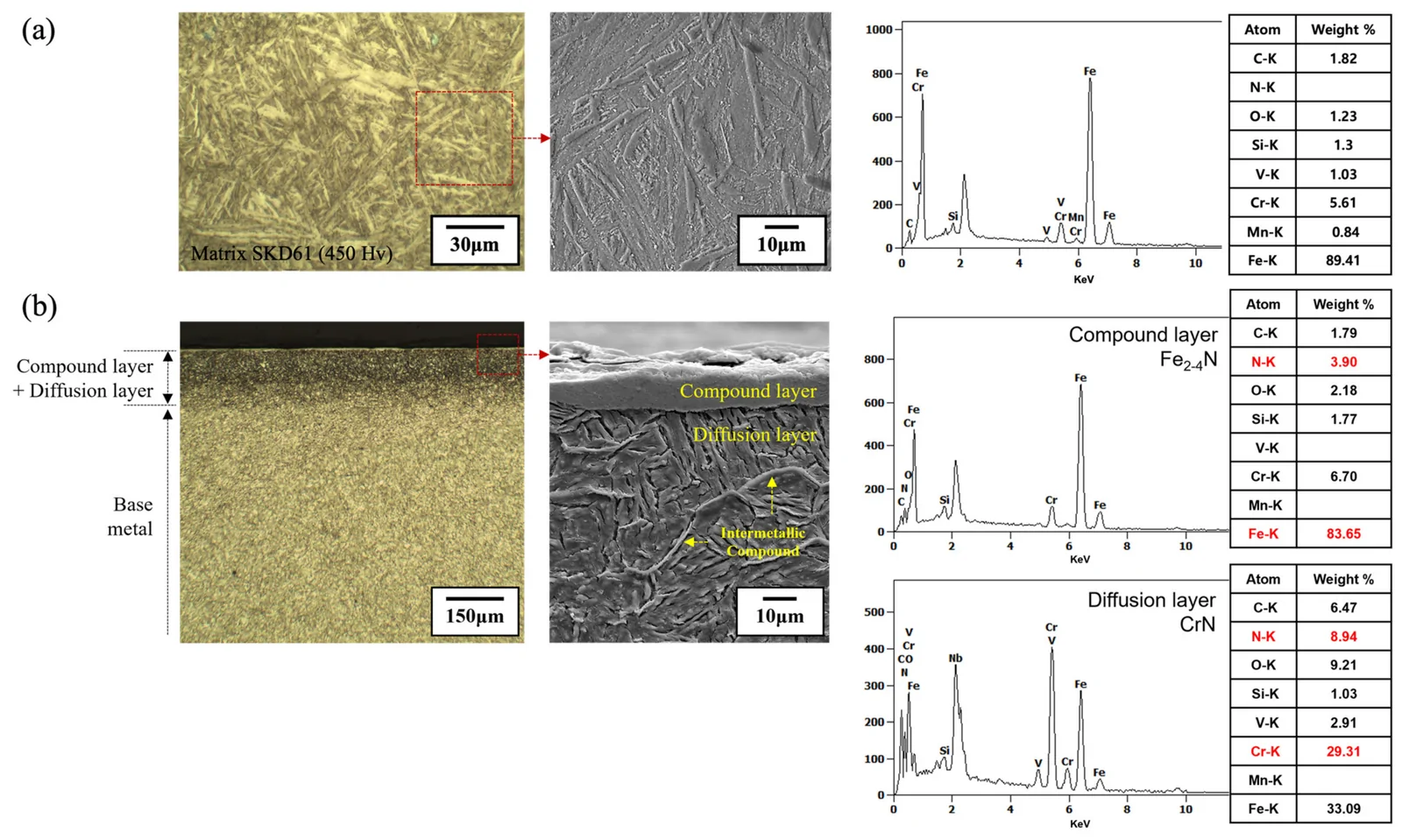
Cross-sectional microstructure characterized by OM, SEM, and EDS: (**a**) as-received; (**b**) after duplex surface treatment. The red dashed boxes indicate the regions shown at higher magnification.

Figure 4 shows representative cross-sectional microstructures of the as-received SKD61 and duplex-treated specimens, as observed by OM and SEM. The as-received SKD61 sample exhibited a QT martensitic microstructure (Figure 4a). The duplex-treated specimens consisted of two layers: a compound layer and diffusion layer (Figure 4b). The compound layer was 2–12 μm thick (Table 5) and exhibited high S and N contents, indicating the formation of Fe_x_N nitride compounds [22,30]. The diffusion layer below the compound layer was 36–147 μm thick. Intermetallic compounds were observed at the grain boundaries, and energy-dispersive X-ray spectroscopy (EDS) analysis confirmed them to be CrN, based on their high Cr and N contents. N preferentially diffuses into the metal along the grain boundaries; therefore, Cr_x_N_y_ intermetallic compounds were considered to have precipitated at these boundaries. The matrix of the diffusion layer showed increased N and S contents compared to those of the as-received specimen.

**Figure 5:**
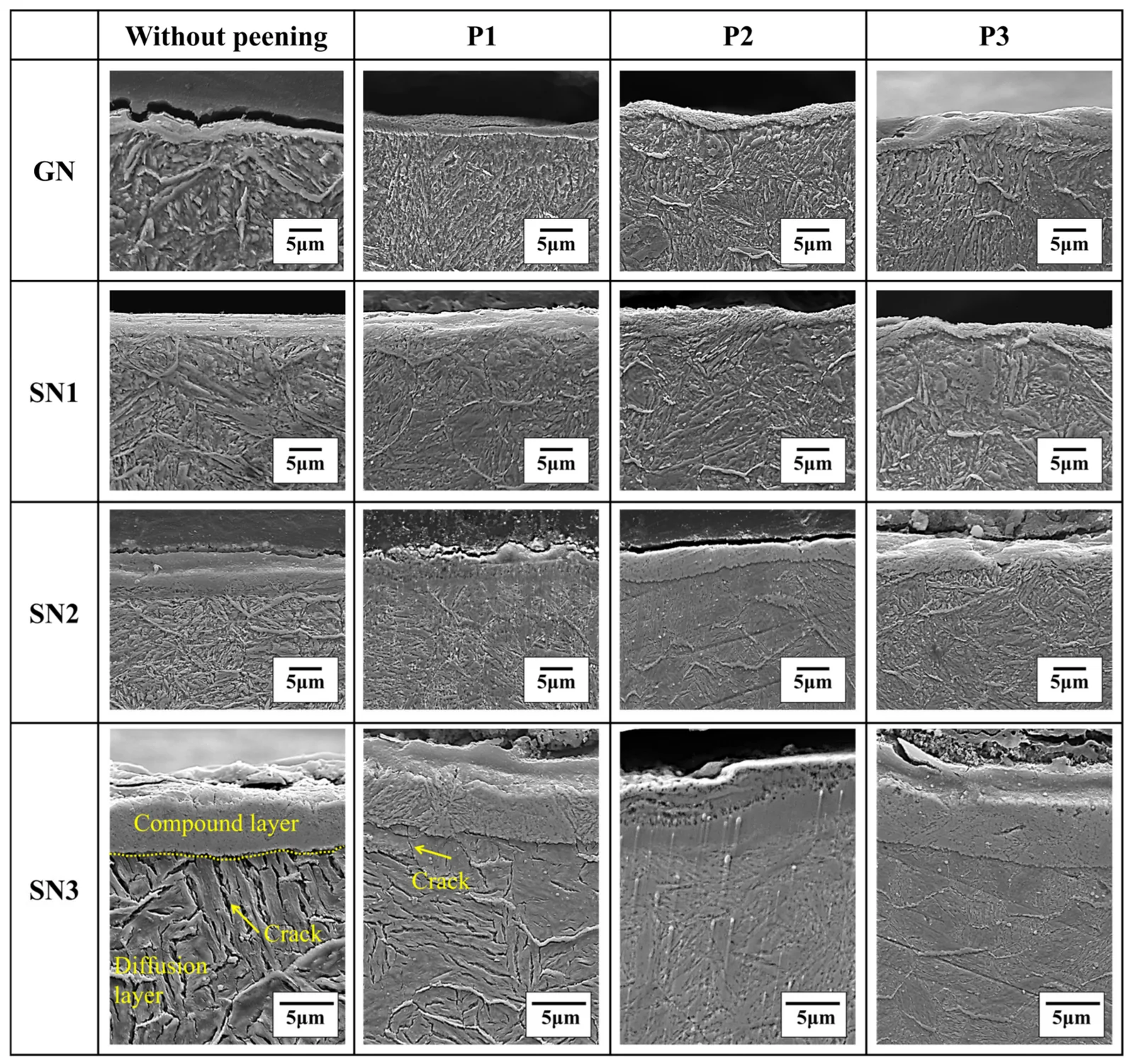
Cross-sectional SEM images of the compound layers of the duplex-surface-treated specimens. The dashed line in the SN3 (without peening) image indicates the boundary between the compound and diffusion layers, and the arrows indicate cracks in the diffusion layer of the SN3 specimens.

**Figure 6:**
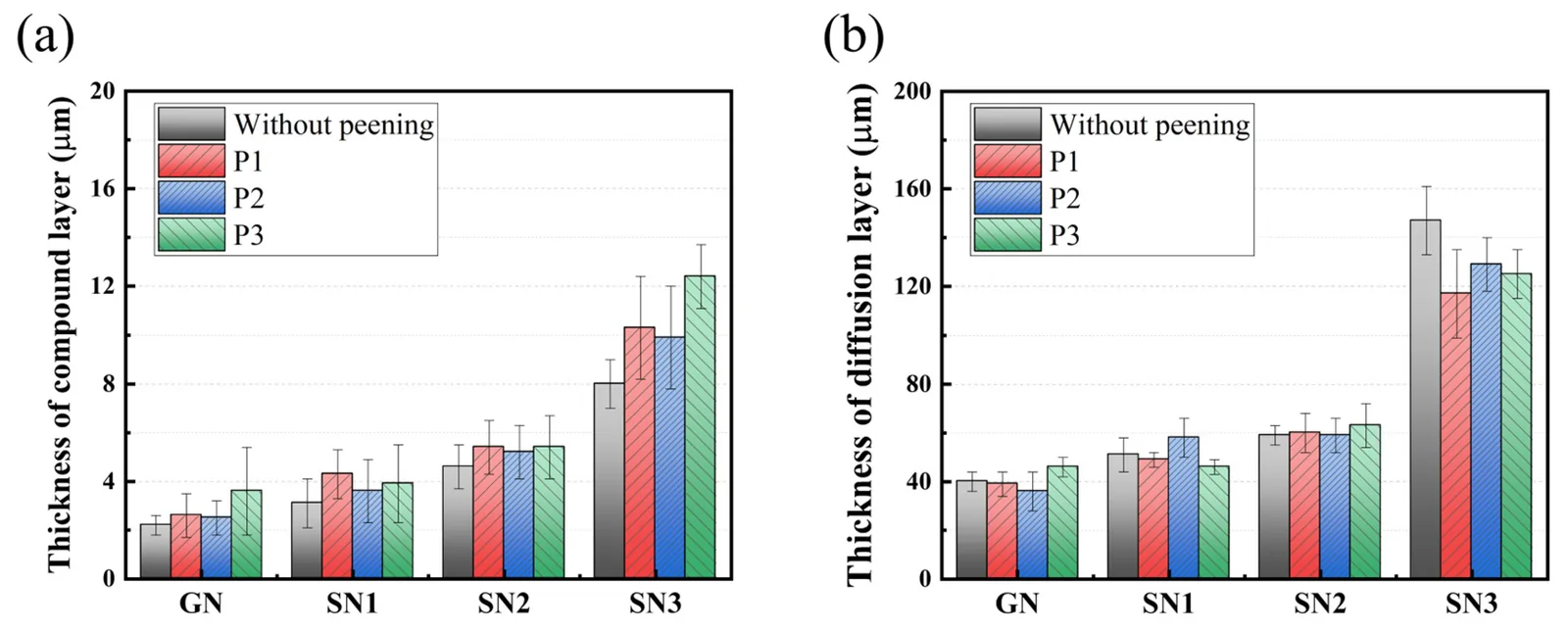
Average thickness of the (**a**) compound and (**b**) diffusion layers of the duplex-surface-treated specimens. Error bars represent the standard deviation of 10 positions per condition.

Figure 5 shows SEM images of the compound layer for specimens subjected to duplex surface treatment under different microshot peening and nitriding conditions. Figure 6 shows the average thicknesses of the compound and diffusion layers, as measured from the images in Figure 5. A comparison of the GN and SN1 specimens treated for the same time showed that the compound layer of SN1 was thicker than that of GN. The thickness of the compound layer increased with increasing sulfonitriding temperature and time from SN1 to SN3. The diffusion-layer thickness also increased slightly for SN1 compared with GN and further increased with increasing sulfonitriding temperature and time from SN1 to SN3. For the microshot-peened specimens, the compound-layer thickness increased slightly relative to that of the non-peened specimens, regardless of the peening parameters. In contrast, peening had no significant effect on diffusion-layer thickness.

Although the effect of the peening parameters was minor, microshot peening performed prior to nitriding appeared to have contributed to the increase in the compound-layer thickness. Microshot peening induced uniform and intense plastic deformation on the outermost surface of the specimen. The resulting localized lattice distortion promoted the formation of subgrains, which increased the grain-boundary density and thereby accelerated atomic diffusion [13]. During the nitriding of the peened specimens, gases such as NH3 and H2S diffused more rapidly into the specimen through lattice dislocations and subgrains, resulting in the formation of a thicker compound layer. The limited effect of microshot peening on the thickness of the diffusion layer is likely because the plastic deformation induced by peening generates a high compressive residual stress only at the outermost surface. As shown in Figure 5 and Figure 6, the duplex-treated specimens exhibited thicker compound layers than those subjected to nitriding alone, which is attributed to the increased dislocation density and grain refinement introduced by peening.

**Figure 7:**
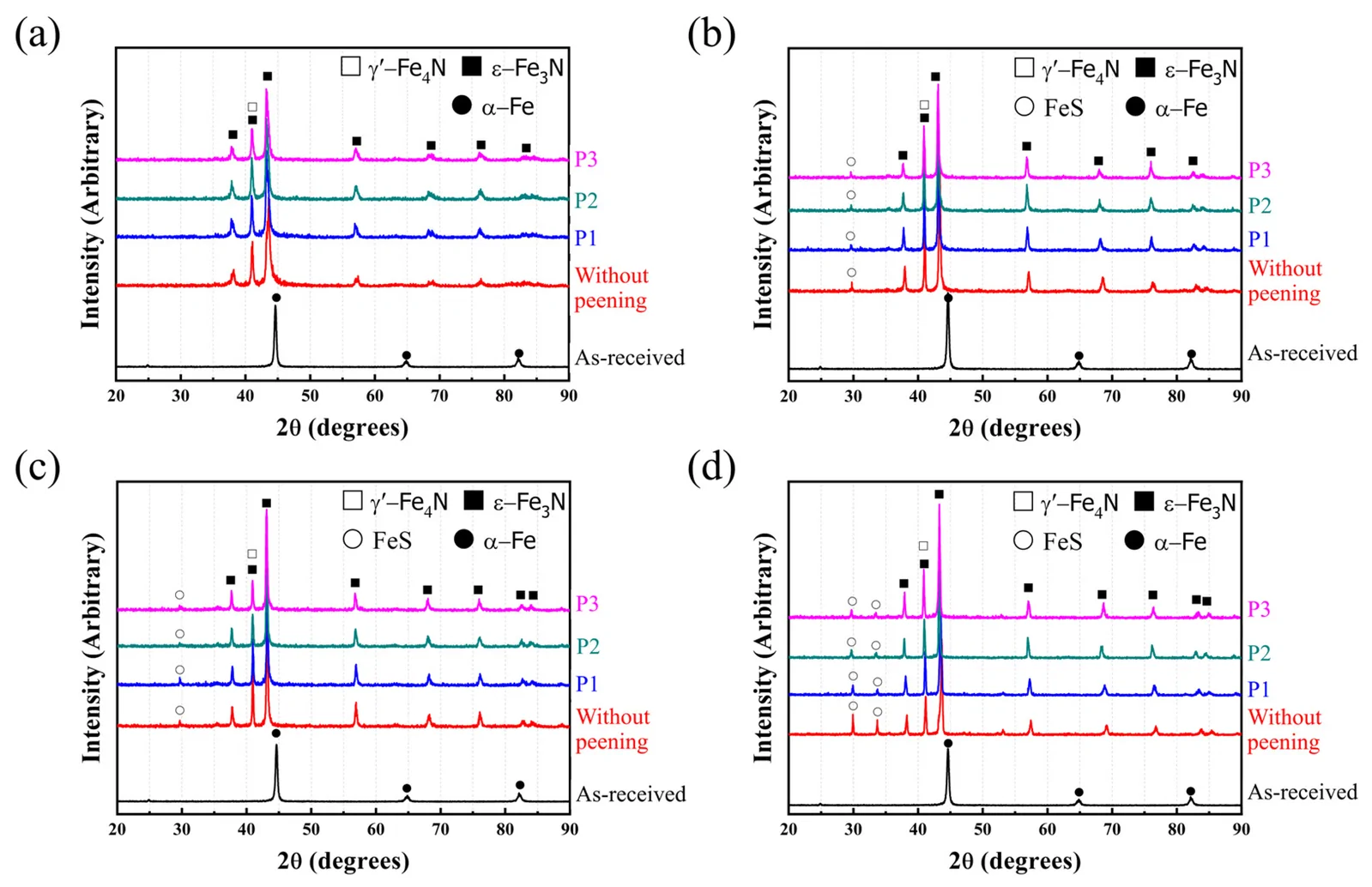
XRD patterns of (**a**) GN, (**b**) SN1, (**c**) SN2, and (**d**) SN3 specimens, without peening and with the P1, P2, and P3 peening conditions (2θ = 20–90°).

Figure 7 shows the XRD patterns of the compound layers of the duplex-treated specimens. In the GN specimens, the γ′-Fe_4_N and ε-Fe_3_N phases were identified. In the SN specimens, FeS reflections were observed together with those of γ′-Fe_4_N and ε-Fe_3_N (Figure 7), and S enrichment of the compound layer was detected by EDS (Figure 4). The FeS reflections became more distinct from SN1 to SN3; however, the phase fractions were not determined quantitatively.

The surface residual stress of the as-received specimen was compressive (−103 MPa) [23]. After nitriding or sulfonitriding of the P2-peened specimens, the surface residual stress was tensile: +181 MPa for P2-GN [23], +162 MPa for P2-SN1, +157 MPa for P2-SN2 and +154 MPa for P2-SN3 (Table 5). Thus, the compressive residual stress introduced by peening was not retained at the surface after nitriding at 450–600 °C, and the compound layers were in tension regardless of the nitriding condition. The residual-stress depth profile through the compound and diffusion layers was not measured.

### 3.2. Properties

Table 5 summarizes the microshot-peened surface roughness prior to nitriding, together with the compound- and diffusion-layer thicknesses and the surface residual stress discussed in Section 3.1. It also summarizes the evaluated hardness, wear resistance, erosion resistance, and heat-checking resistance.

#### 3.2.1. Hardness

**Figure 8:**
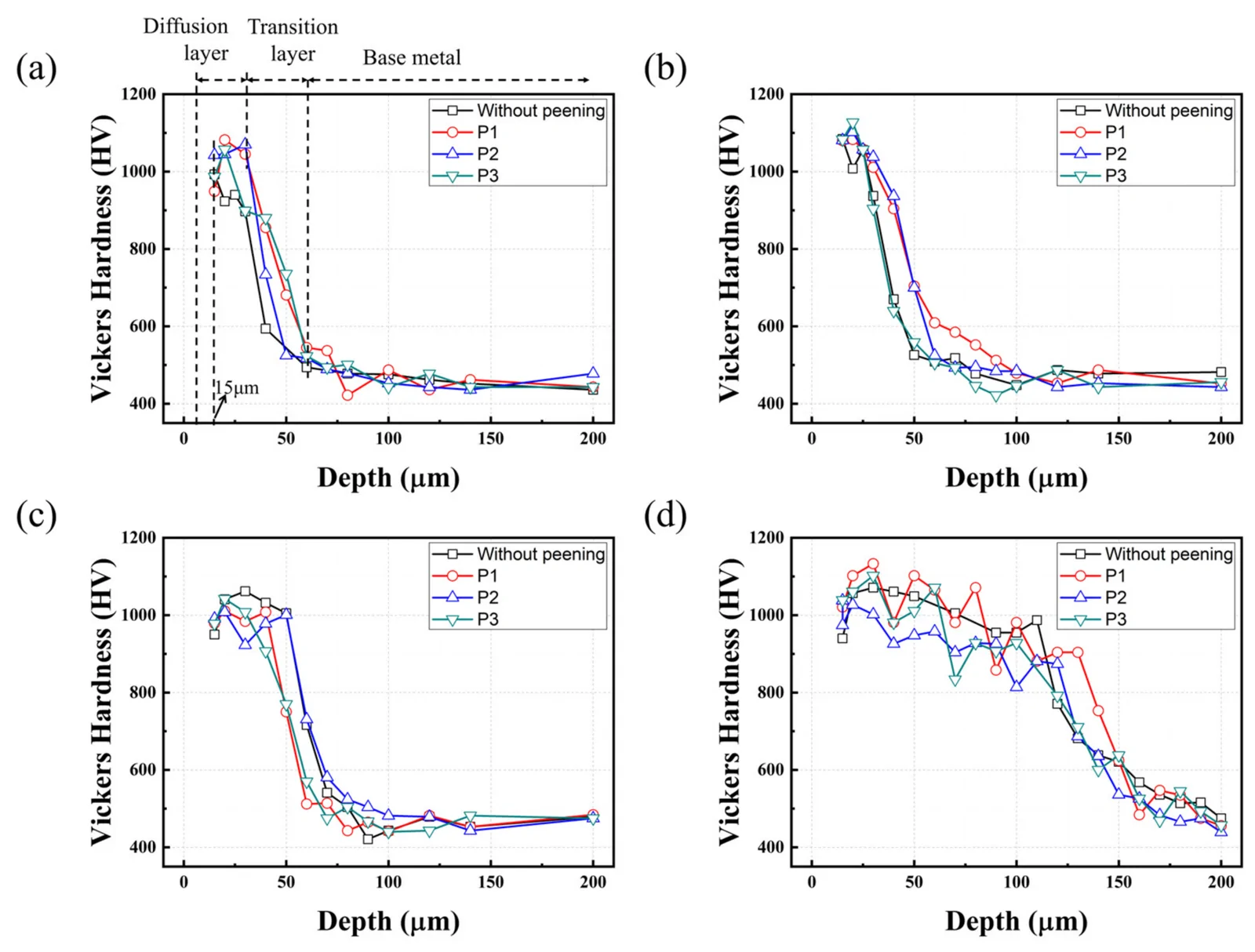
Vickers hardness (HV0.1) depth profiles of the (**a**) GN, (**b**) SN1, (**c**) SN2 and (**d**) SN3 specimens. The first indentation was made 15 μm below the surface (one indentation per depth).

Figure 8 shows the hardness depth profiles measured on the cross-sections of the duplex-treated specimens. The near-surface hardness values (measured 15 μm below the surface) of the GN and SN specimens increased to approximately 1060 HV, improving by approximately 135% on the as-received specimen (450 HV). No significant effects of the process parameters were observed. The diffusion layer exhibited high hardness owing to the presence of CrN at the grain boundaries, whereas the transition zone between the diffusion layer and base material showed a sharp decrease in hardness because CrN did not form in this region. Although the hardness of the near-surface diffusion layer remained largely unchanged with increasing sulfonitriding time and temperature, from SN1 to SN3, the high-hardness diffusion layer thickened under these conditions.

#### 3.2.2. Wear Resistance

**Figure 9:**
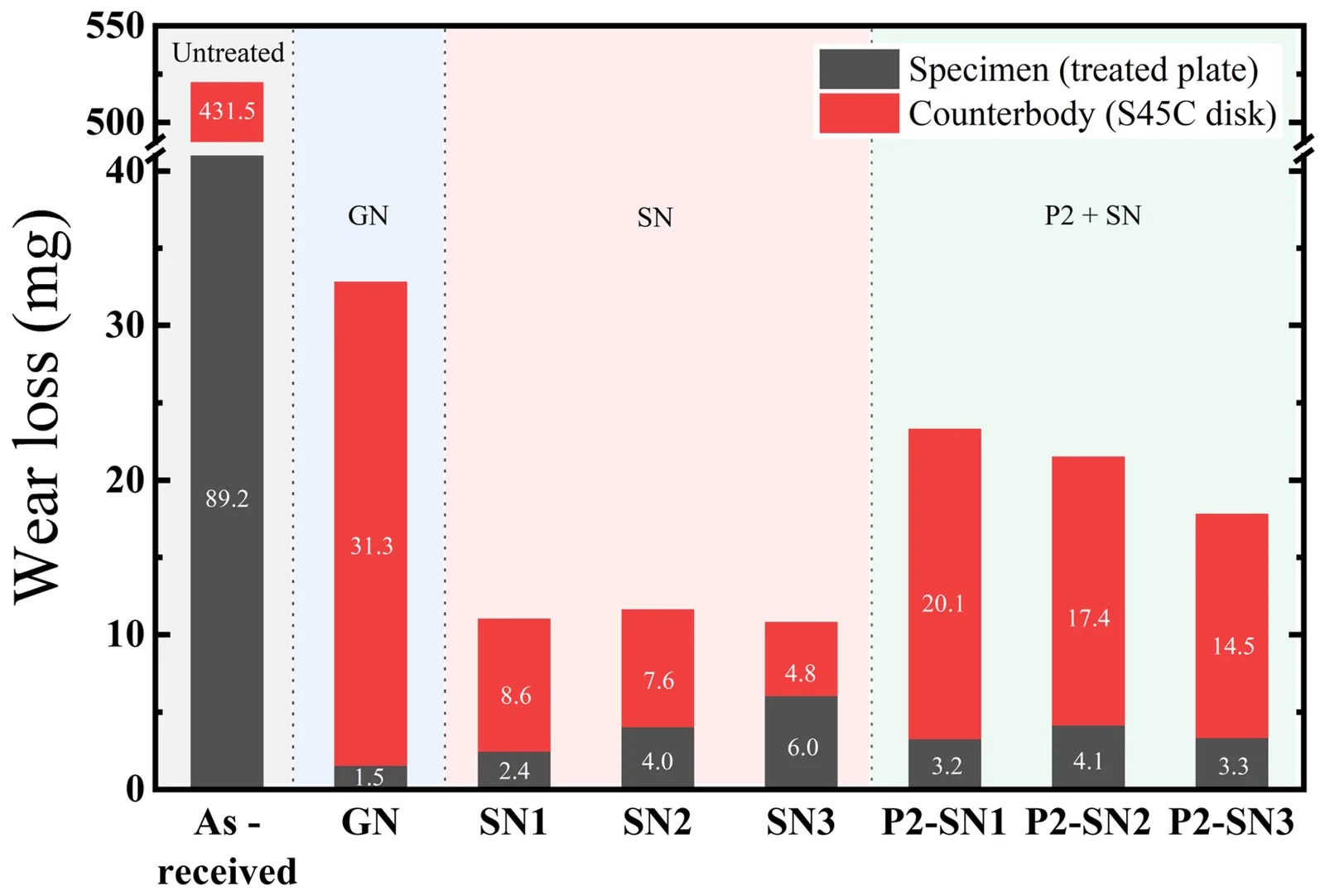
Wear losses of the specimens (plates) and the S45C counterbodies (disks) for each process condition, stacked in each bar; the numbers give the individual wear losses. One test was performed for each condition; the GN-P2 specimen was not tested.

Because the surface topography produced by processing, such as tailored surface texturing, affects the wear behavior of contacting surfaces [31], and the wear mechanism of a tribo-pair can differ between contact states [32], the wear losses of both the treated specimen and the counterbody were evaluated in this study. Figure 9 shows the wear losses of the treated specimens (plates) and of the S45C counterbodies (disks). All nitrided specimens showed specimen wear losses 15–60 times smaller than that of the as-received specimen (1.5–6.0 mg vs. 89.2 mg), owing to the hard compound and diffusion layers. Among them, the GN specimen showed the lowest specimen wear loss (1.5 mg), and the specimen wear loss of the SN specimens increased from SN1 (2.4 mg) to SN3 (6.0 mg). In contrast, the wear loss of the counterbody was 4–7 times smaller against the SN specimens (4.8–8.6 mg) than against the GN specimen (31.3 mg), and it decreased from SN1 to SN3. Consequently, the total wear loss of the tribo-pair for the SN specimens (10.8–11.6 mg) was approximately one-third that of the GN specimen (32.8 mg). These results are consistent with the role of FeS reported in previous studies: the soft FeS-containing surface layer is worn or transferred during sliding and reduces adhesion and abrasion of the counterface [19,20,21,22,33,34] so that the treated surface itself loses slightly more mass, while the counterbody and the tribo-pair as a whole wear less. Because the friction coefficient and the worn surfaces were not analyzed, this mechanism could not be confirmed directly. Prior P2 peening increased the counterbody wear loss (14.5–20.1 mg vs. 4.8–8.6 mg), most likely because the rougher peened surface (Rz 18.6–19.5 μm before nitriding) abraded the softer S45C disk, whereas its effect on the specimen wear loss was not consistent.

Mechanical pretreatments such as burnishing or surface nanocrystallization before sulfonitriding or sulfurizing have been reported to change the phase composition of the sulfide layer and its friction and wear behavior [24,25], which is consistent with the different wear behavior of the peened and non-peened SN specimens in this study. The surface roughness after nitriding was not measured; because the compound layer (2–12 μm) follows the peened surface profile, it is expected to be of the same order as that before nitriding, but this was not verified.

#### 3.2.3. Erosion Resistance

**Figure 10:**
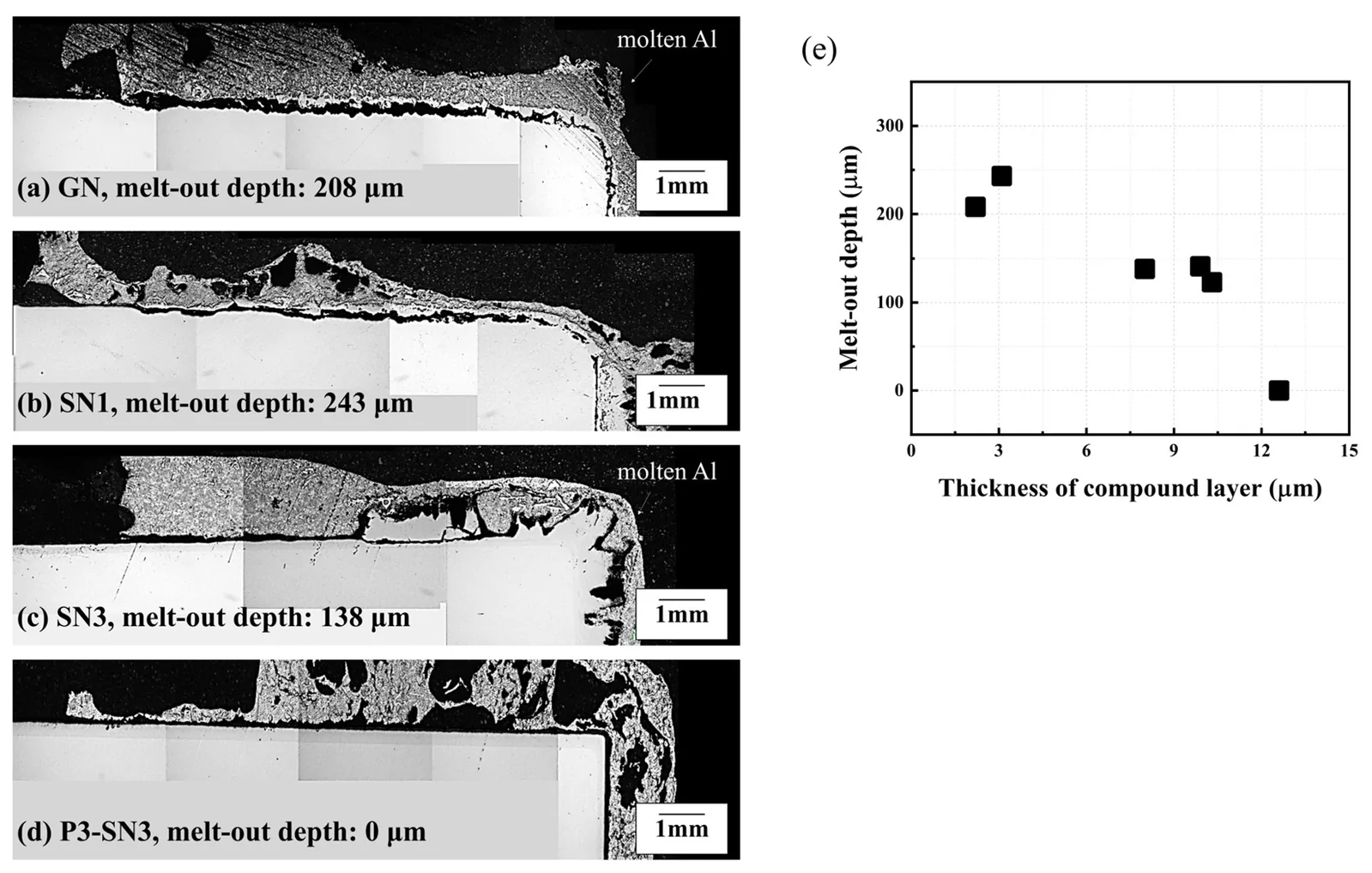
(**a**–**c**) OM images of specimen cross-sections after immersion in molten aluminum, (**d**) OM image of the P3-SN3 specimen cross-section, and (**e**) melt-out depth as a function of compound-layer thickness (Al erosion resistance).

Figure 10a–c show OM images of specimen cross-sections after immersion in molten aluminum at 700 °C for 43 h, which were used to evaluate the erosion resistance. When the GN and SN1 specimens treated for the same time were compared, no notable differences in the erosion resistance were observed. This suggests that, unlike its effect on wear resistance, the formation of the FeS phase in the compound layer plays a minor role in erosion resistance. The melt-out depth decreased with increasing sulfonitriding time and temperature from SN1 to SN3. Additional erosion-resistance tests were performed on the duplex-treated specimens with thicker compound layers (P1-SN3, P2-SN3, and P3-SN3). Figure 10d shows an OM image of the cross-section of the P3-SN3 specimen after 43 h of immersion. Figure 10e shows the variation in the melt-out depth as a function of the compound-layer thickness. The melt-out depth decreased with increasing compound-layer thickness. These results indicate that the compound-layer thickness is a key factor governing erosion resistance.

#### 3.2.4. Heat-Checking Resistance

**Figure 11:**
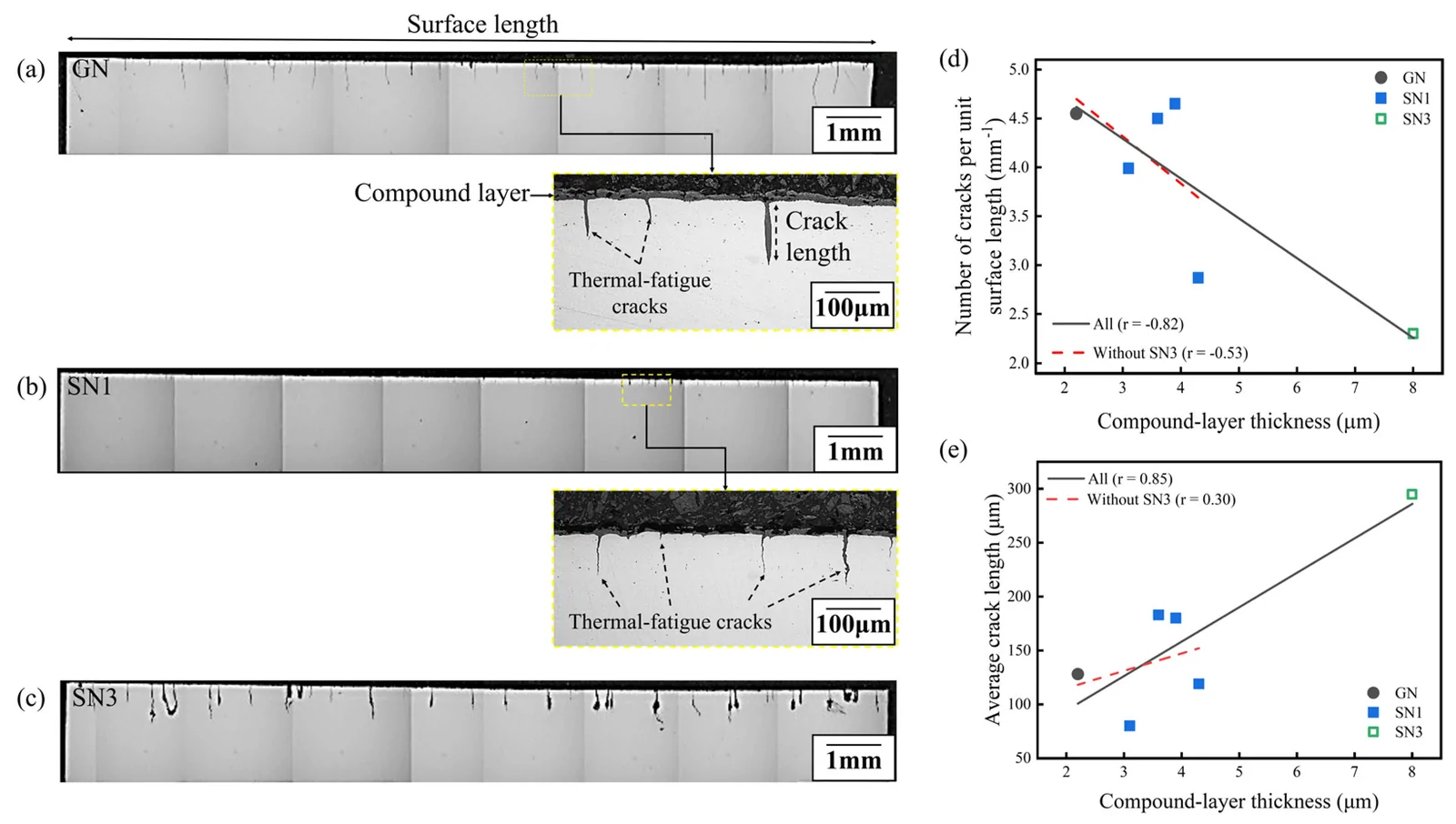
(**a**–**c**) Cross-sectional OM images of thermal-fatigue cracks after 3000 cycles, (**d**) number of cracks per unit surface length, and (**e**) average crack length as functions of the compound-layer thickness. One specimen was tested for each condition, and all cracks along 61–69 mm of the cross-section (154–306 cracks per specimen) were measured. Solid lines: all specimens; dashed lines: without SN3.

Figure 11a–c show OM images of the specimen cross-sections after 3000 cycles of the thermal-shock-fatigue test. Cracks were observed on the specimen surfaces because of repeated thermal-shock fatigue. A comparison between the GN (Figure 11a) and SN1 (Figure 11b) specimens treated for the same time showed that the crack length in the SN1 specimen was shorter. This suggests that the FeS phase in the compound layer may have resisted crack propagation. It should be noted, however, that the distribution of FeS in the compound layer and its interaction with the cracks were not observed by SEM in this study. The following explanation is therefore a hypothesis based on the literature. Secondary phases dispersed in a brittle matrix can increase toughness by deflecting or pinning the crack tip [35,36,37], and for ductile inclusions, the stress-intensity factor inside the inclusion is lower than in the matrix; thus, cracks can be arrested at the inclusion boundary [38]. Because FeS has a low shear strength, it may delay crack propagation in a similar manner. Cross-sectional SEM and EDS mapping of the crack paths are needed to verify this. Moreover, the cross-sectional observations showed longer cracks in the SN3 specimen than in the SN1 specimen (Figure 11c); the SN2 series was not included in the thermal-shock-fatigue tests. The same thermal-shock-fatigue tests were performed on specimens in which the three peening conditions (P1, P2, and P3) were additionally applied to the SN1 condition, which exhibited the best thermal-shock-fatigue performance (P1-SN1, P2-SN1, and P3-SN1), to examine the correlation between the compound-layer thickness and crack behavior and to obtain a broader range of compound-layer thicknesses. Table 5 summarizes the quantified average crack length, maximum crack length and number of cracks per unit length as evaluation parameters for the entire test set, including the GN, SN1 and SN3 specimens and the newly added P1/P2/P3-SN1 specimens.

Figure 11d,e show the number of cracks per unit length and the average crack length as functions of the compound-layer thickness. Specimens with thicker compound layers tended to show fewer but longer cracks (r = −0.82 and 0.85, respectively). However, these trends depend largely on the SN3 specimen; without it, the correlation coefficients decrease to −0.53 and 0.30. In addition, SN3 was sulfonitrided at a higher temperature for a longer time than the other specimens, so the effect of the compound-layer thickness cannot be separated from that of the process conditions, and only one specimen was tested for each condition. The trends should therefore be regarded as preliminary, and repeated tests, including the SN2 series, which was not tested in this study, are needed to confirm them. In all specimens, the average crack length (80–295 μm) exceeded the diffusion-layer thickness (40–147 μm), indicating that the cracks propagated through the diffusion layer. The duplex-treated specimens exhibited thicker diffusion layers (Figure 6), with hardness values similar to those of the SN1 and SN2 specimens (Figure 8). However, cracks were observed within the diffusion layers of the SN3 specimens, as shown in Figure 5. This was likely because the nitride phases became excessively coarsened owing to the prolonged high-temperature sulfonitriding treatment. In the SN3 specimens, which were sulfonitrided at high temperatures for an extended time, the cracks generated during the thermal-shock-fatigue test propagated more readily along the pre-existing cracks formed in the diffusion layer during sulfonitriding. Although a thick compound layer appears to suppress crack initiation, the processing parameters used to form a thick compound layer may adversely affect the thermal-shock-fatigue life from a crack-propagation perspective.

This opposing trend may be interpreted in multiple ways. In this study, the near-surface hardness itself did not vary substantially with increasing compound-layer thickness (Section 3.2.1); however, the crack-initiation resistance improved with thickness. This suggests that factors other than hardness, such as the distribution of defects within the compound layer, may have influenced the crack-initiation behavior; the surface residual stress was similar for all P2-peened specimens (Table 5) and therefore, does not explain these differences. For crack propagation, compared with the matrix, the compound layer is composed of relatively brittle ceramic-like phases (e.g., Fe_x_N); therefore, it may possess relatively low fracture toughness, which is considered a plausible reason for the propagation of cracks within the compound layer. Strategies such as the SN treatment, which forms a ductile phase such as FeS within the compound layer, may have helped compensate for this reduced crack-propagation resistance. However, because the fracture toughness and defect density of the compound layer were not directly measured in this study, this interpretation remains tentative and warrants further verification.

These results align with a recently reported cooling-cycle-based thermal-shock-fatigue life-prediction model for die-casting mold steels, which indicates that crack initiation and propagation should be modeled as separate stages governed by different mechanisms [4,39]. Therefore, a multistage life-prediction model that considers both crack-initiation and -propagation resistances should be applied in future work to improve the thermal-shock-fatigue life by optimizing the compound-layer thickness.

### 3.3. Nondestructive Evaluation

**Figure 12:**
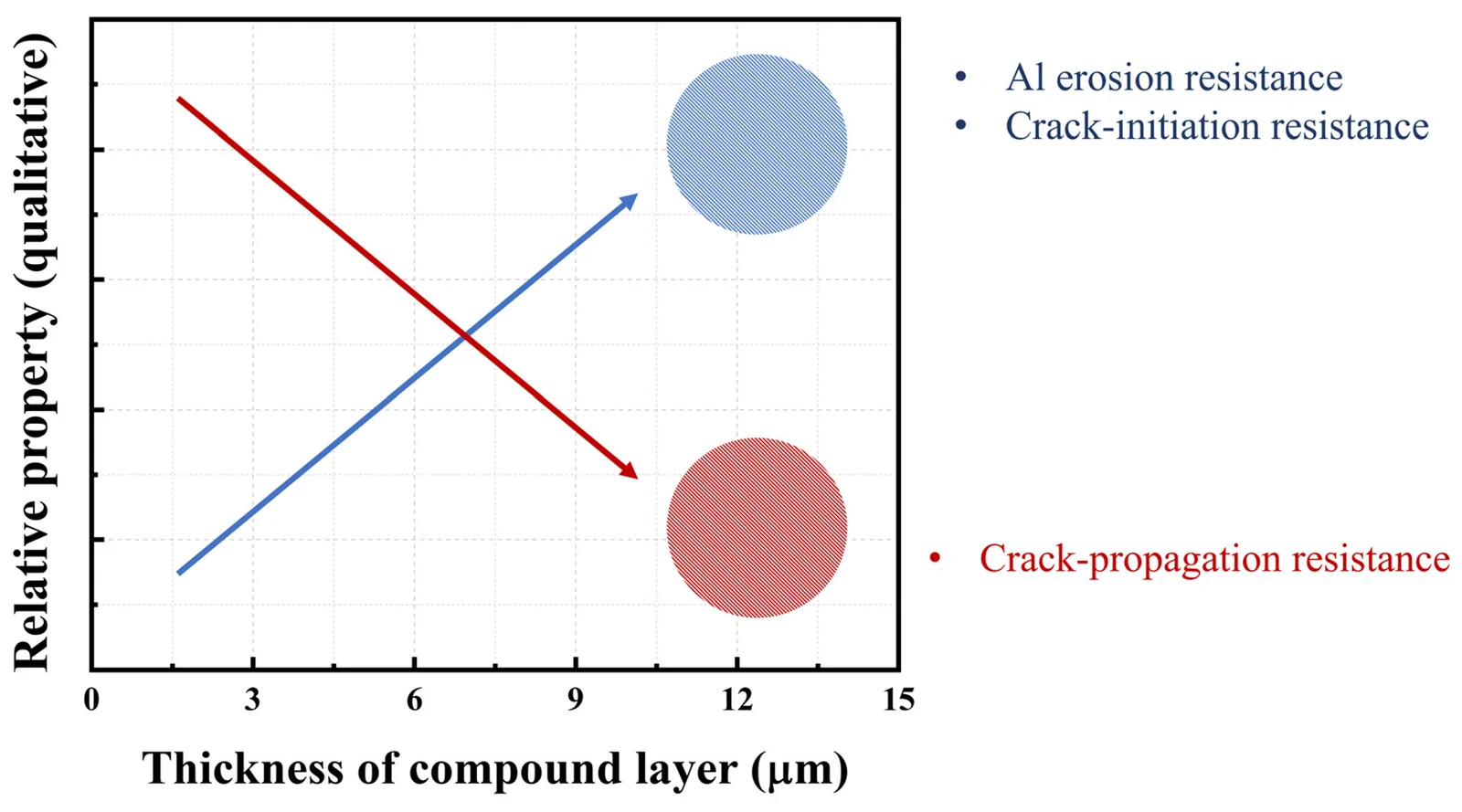
Schematic summary of the qualitative trends of the reliability properties with the compound-layer thickness.

Figure 12 schematically summarizes the qualitative trends in erosion resistance, crack-initiation resistance, and crack-propagation resistance as functions of the sulfonitrided compound-layer thickness. Several key properties required for die-casting molds, particularly the erosion resistance and the thermal-shock-fatigue behavior, depend on the compound-layer thickness of the duplex-treated SKD61. The microstructures of magnetic materials such as SKD61 can be evaluated nondestructively using magnetic sensors [40]. In this study, the compound-layer thickness of the duplex-treated SKD61 specimens was nondestructively evaluated using Barkhausen-noise analysis.

#### 3.3.1. Correlation Between Compound-Layer Thickness and Magnetization

**Figure 13:**
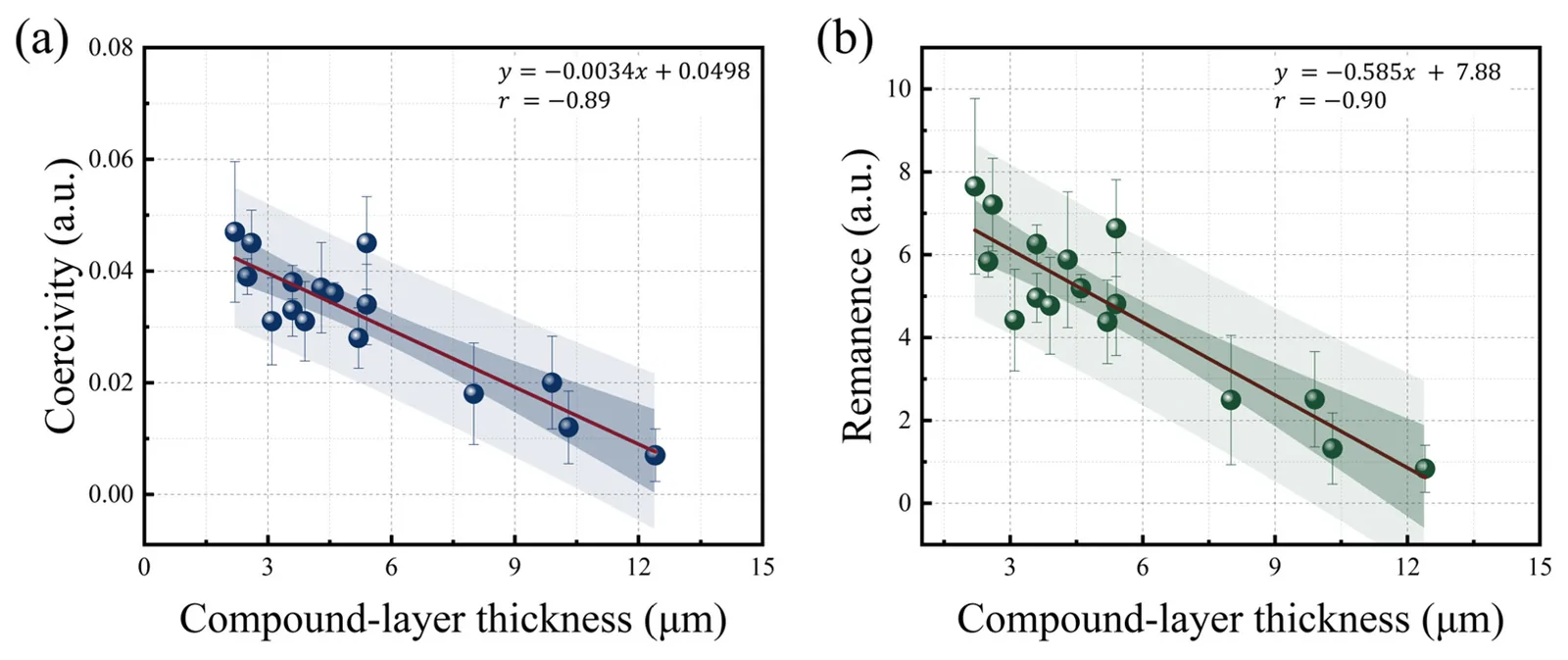
(**a**) Coercivity and (**b**) remanence as functions of the compound-layer thickness for all duplex-treated specimens. The lines are linear regressions (coercivity: y = −0.0034x + 0.0498, r = −0.89; remanence: y = −0.585x + 7.88, r = −0.90).

Barkhausen-noise analysis was applied to all specimens with different compound-layer thicknesses to measure the coercivity and remanence, analyzing the correlation between the compound-layer thickness and magnetic behavior of the specimens. Table 6 summarizes the coercivity and remanence values of each specimen. The coercivity and remanence of the as-received SKD61 were 0.094 ± 0.0091 a.u. and 19.29 ± 2.25 a.u., respectively. Figure 13a,b show the variation in coercivity and remanence as a function of the compound-layer thickness. Both the coercivity and remanence decreased linearly with increasing compound-layer thickness (r = −0.89 and −0.90, respectively).

All duplex-treated specimens exhibited lower coercivity and remanence values than those of the as-received SKD61. Although the γ′-Fe_4_N and ε-Fe_3_N phases are ferromagnetic at room temperature [41,42], the compound layer differs from the martensitic matrix in crystal structure, magnetization and microstructure, and in the SN specimens, it also contains FeS (XRD and EDS). In Barkhausen-noise measurements of nitrided steels, the compound layer has been reported to contribute little to the detected signal and to act as a gap that attenuates the signal from the underlying steel [43]. As the compound layer becomes thicker, the Barkhausen-active steel beneath it is magnetized less effectively and lies farther from the sensor, which is a plausible reason for the decrease in both parameters (Figure 13). The high hardness and residual stress of the diffusion layer may also affect the signal [40]; separating these contributions, for example, by measurements after stepwise removal of the compound layer, remains a subject for future work.

These results suggest that the compound-layer thickness can be estimated by measuring these two parameters.

#### 3.3.2. Nondestructive Assessment of Compound-Layer Thickness

Because the compound-layer thickness governs the erosion resistance and affects the thermal-shock-fatigue behavior (Figure 10e and Figure 11d,e), a nondestructive method for checking this thickness after treatment is useful for the quality control of duplex-treated molds. The linear relationships in Figure 13 (r = −0.89 for coercivity and −0.90 for remanence) can serve as calibration curves for estimating the compound-layer thickness of SKD61-based steel treated under the conditions used in this study. Previous studies have predicted the case depth and surface hardness of nitrided or hardened steels from Barkhausen-noise signals [18,26,27]; the present results show that the compound-layer thickness of sulfonitrided steel can also be assessed in this way. However, the calibration was obtained from as-treated specimens of a single steel grade measured with one instrument setting. Applying it to molds after service, where wear, erosion, oxidation and thermal cracking also change the surface, requires further validation with service-exposed specimens.

## 4. Conclusions

This study examined duplex surface treatments combining microshot peening (P1–P3) with gas nitriding (GN) or sulfonitriding (SN1–SN3) of SKD61-based die-casting mold steel. The main conclusions are as follows.

(1) Both GN and SN formed compound layers of γ′-Fe_4_N and ε-Fe_3_N; in the SN specimens, FeS was additionally identified by XRD and confirmed by the S enrichment detected by EDS. The compound-layer thickness (2–12 μm) increased from SN1 to SN3 and increased slightly with prior peening, which is attributed to faster nitrogen diffusion through the deformed surface; peening scarcely affected the diffusion-layer thickness.
(2) The near-surface hardness, measured 15 μm below the surface, was approximately 1060 HV for all GN and SN specimens and was not significantly affected by the process parameters. The hardness of the compound layer itself was not measured.
(3) All nitrided specimens wore 15–60 times less than did the as-received specimen. The GN specimen showed the lowest specimen wear loss, whereas sulfonitriding reduced the wear loss of the S45C counterbody by a factor of 4–7 and that of the tribo-pair by about two-thirds; the specimen wear loss of the SN specimens increased slightly from SN1 to SN3. This behavior is consistent with a soft FeS-containing surface layer that is consumed during sliding and protects the counterface, although the friction coefficient and worn surfaces were not analyzed. Prior peening increased the counterbody wear, most likely because of the higher surface roughness, and did not consistently change the specimen wear; the wear benefit of the duplex treatment is therefore due to sulfonitriding, not to peening.
(4) The melt-out depth in molten aluminum decreased with increasing compound-layer thickness, whereas GN and SN1 showed similar erosion resistance. The compound-layer thickness, rather than FeS, governs the erosion resistance.
(5) In the thermal-shock-fatigue tests, specimens with thicker compound layers tended to show fewer but longer cracks. Because this trend relies largely on the SN3 specimen, which was treated at a higher temperature for a longer time, it is preliminary; it indicates that crack initiation and propagation should be considered separately when the compound-layer thickness is optimized.
(6) The Barkhausen-noise coercivity and remanence decreased with increasing compound-layer thickness (r = −0.89 and −0.90), which is attributed mainly to the reduced contribution of the steel beneath the compound layer. These parameters can therefore be used to assess the compound-layer thickness nondestructively after treatment, for example, for quality control. Applying this approach to molds in service requires validation with service-exposed specimens, and the separate effects of the compound layer, the diffusion layer and the residual stress on the signal remain to be clarified.

## Figures and Tables

**Table 1:** Chemical composition of the FDAC steel (ladle analysis from the manufacturer’s test certificate, wt%).

C	Si	Mn	P	S	Cr	Mo	V	Fe
0.37	0.84	0.68	0.027	0.13	5.08	1.21	0.32	Bal.

**Table 2:** Microshot-peening process conditions.

Parameter	P1	P2	P3
Ball material	Steel		
Spray distance (mm)	100–250		
Ball size (mm)	0.3	0.5	0.7
Spray time (s)	60	180	300
Spray pressure (MPa)	3	5	7
Roughness	Rz 14	Rz 18	Rz 22

**Table 3:** GN and SN process conditions.

Variable	GN	SN1	SN2	SN3
Time (h)	10	10	15	20–25
Temperature (°C)	500–560	450–530	450–550	530–600
Gas	NH_3_	NH_3_, H_2_S	NH_3_, H_2_S	NH_3_, H_2_S

**Table 4:** Wear-test conditions for duplex-surface-treated specimens.

Parameters	Ranges
Tester	Plint TE77 (reciprocating)
Fixed specimen	Duplex-treated plate
Counterbody	S45C disk, ø12 mm (area contact)
Applied load	100 N
Stroke length	±15 mm
Frequency	2 Hz
Friction time	3600 s
Lubrication	None (25 ± 2 °C, 60 ± 1% RH)
Number of tests	One per condition
Measured quantity	Weight loss of specimen and counterbody

**Table 5:** Summary of microstructural characteristics and reliability properties of duplex-surface-treated SKD61 specimens.

Duplex Surface Treatment		Roughness After Peening ^a^ (Rz, μm)	Compound-Layer Thickness ^b^ (μm)	Diffusion-Layer Thickness ^b^ (μm)	Surface Residual Stress ^f^ (MPa)	Wear Loss ^c^ (mg)		Melt-Out Depth (μm)	Thermal-Fatigue Cracks ^d^		
Nitriding	Peening					Specimen	Counter-Body		Average Crack Length (μm/Crack)	Number of Cracks per Unit Length (mm^−1^)	Maximum Crack Length (mm)
As-received		–	–	–	−103 [23]	89.2	431.5	n.t.	n.t.	n.t.	n.t.
GN	–	–	2.2 ± 0.4	40 ± 4	n.t.	1.5	31.3	208	128	4.55	1.1
	P1	14.8	2.6 ± 0.9	39 ± 5	n.t.	n.t.	n.t.	n.t.	n.t.	n.t.	n.t.
	P2	19.3	2.5 ± 0.7	36 ± 8	+181 [23]	n.t.	n.t.	n.t.	n.t.	n.t.	n.t.
	P3	22.8	3.6 ± 1.8	46 ± 4	n.t.	n.t.	n.t.	n.t.	n.t.	n.t.	n.t.
SN1	–	–	3.1 ± 1.0	51 ± 7	n.t.	2.4	8.6	243	80	3.99	0.1
	P1	14.5	4.3 ± 1.0	49 ± 3	n.t.	n.t.	n.t.	n.t.	119	2.87	1.0
	P2	19.5	3.6 ± 1.3	58 ± 8	+162	3.2	20.1	n.t.	183	4.50	1.4
	P3	23.2	3.9 ± 1.6	46 ± 3	n.t.	n.t.	n.t.	n.t.	180	4.65	2.2
SN2	–	–	4.6 ± 0.9	59 ± 4	n.t.	4.0	7.6	n.t.	n.t.	n.t.	n.t.
	P1	15.2	5.4 ± 1.1	60 ± 8	n.t.	n.t.	n.t.	n.t.	n.t.	n.t.	n.t.
	P2	18.6	5.2 ± 1.1	59 ± 7	+157	4.1	17.4	n.t.	n.t.	n.t.	n.t.
	P3	23.2	5.4 ± 1.3	63 ± 9	n.t.	n.t.	n.t.	n.t.	n.t.	n.t.	n.t.
SN3	–	–	8.0 ± 1.0	147 ± 14	n.t.	6.0	4.8	138	295	2.30	1.1
	P1	14.6	10.3 ± 2.1	117 ± 18	n.t.	n.t.	n.t.	123	n.t.	n.t.	n.t.
	P2	19.0	9.9 ± 2.1	129 ± 11	+154	3.3	14.5	141	n.t.	n.t.	n.t.
	P3	23.0	12.4 ± 1.3	125 ± 10	n.t.	n.t.	n.t.	0 ^e^	n.t.	n.t.	n.t.

^a^ Measured after peening and before nitriding. ^b^ Mean ± standard deviation of 10 positions in the cross-sectional SEM image of each condition. ^c^ One test per condition; specimen = duplex-treated plate, and counterbody = S45C disk. ^d^ One specimen per condition; all cracks along 61–69 mm of the cross-section (154–306 cracks per specimen). ^e^ No measurable melt-out. ^f^ XRD sin^2^ψ method; positive values indicate tension. –: not applicable; n.t.: not tested.

**Table 6:** Coercivity and remanence of the as-received and duplex-surface-treated SKD61 specimens (mean ± standard deviation of five measurements per specimen).

Duplex Surface Treatment		Coercivity (a.u.)	Remanence (a.u.)
Nitriding	Peening		
As-received		0.094 ± 0.0091	19.29 ± 2.25
GN	–	0.047 ± 0.0126	7.65 ± 2.12
	P1	0.045 ± 0.0059	7.21 ± 1.12
	P2	0.039 ± 0.0032	5.83 ± 0.37
	P3	0.038 ± 0.0030	6.33 ± 0.56
SN1	–	0.031 ± 0.0078	4.42 ± 1.23
	P1	0.037 ± 0.0081	5.88 ± 1.64
	P2	0.033 ± 0.0047	4.96 ± 0.59
	P3	0.031 ± 0.0071	4.77 ± 1.17
SN2	–	0.036 ± 0.0019	5.19 ± 0.33
	P1	0.045 ± 0.0083	6.64 ± 1.17
	P2	0.028 ± 0.0054	4.38 ± 1.01
	P3	0.034 ± 0.0072	4.81 ± 1.24
SN3	–	0.018 ± 0.0091	2.49 ± 1.56
	P1	0.012 ± 0.0065	1.32 ± 0.86
	P2	0.020 ± 0.0083	2.51 ± 1.15
	P3	0.007 ± 0.0047	0.83 ± 0.57

Measured with a Rollscan 300 analyzer (125 Hz, 3 Vpp, 70–200 kHz); values are given in the relative units of the analyzer.

## Data Availability

The data presented in this study are available on request from the corresponding authors.

## Abbreviations

The following abbreviations are used in this manuscript:

EDS	Energy-dispersive X-ray spectroscopy
GN	Gas nitriding
HV	Vickers hardness
JIS	Japanese Industrial Standards
KS	Korean Industrial Standards
OM	Optical microscopy
P1–P3	Microshot peening conditions 1–3
QT	Quenched and tempered
Rz	Maximum height of the roughness profile
SEM	Scanning electron microscopy
SN	Sulfonitriding
SN1–SN3	Sulfonitriding conditions 1–3
XRD	X-ray diffraction
